# Evaluation of oyster mushroom (*Pleurotus ostreatus*)-derived anthraquinone on the induction of apoptosis and suppression of MMP-2 and MMP-9 expression in breast cancer cells

**DOI:** 10.7150/ijms.93334

**Published:** 2024-04-08

**Authors:** Bindhu Jayaprakash, Ashwin Raj Suresh, Rekha Thiruvengadam, Naiyf S. Alharbi, Shine Kadaikunnan, Sathianarayanan Sankaran, Muthu Thiruvengadam, Rethinam Senthil, Baskar Venkidasamy

**Affiliations:** 1 Department of Biotechnology, Sri Shakthi Institute of Engineering & Technology, Coimbatore 641062, India.; 2 Department of Biotechnology, Bannari Amman Institute of Technology, Sathyamangalam 638401, India.; 3 Center for Global Health Research, Saveetha Medical College, Saveetha Institute of Medical and Technical Sciences (SIMATS), Saveetha University, Chennai 600077, India.; 4 Department of Botany and Microbiology, College of Science, King Saud University, P. O. Box 2455, Riyadh 11451, Saudi Arabia.; 5 Department of Pharmaceutical Chemistry, NGSM Institute of Pharmaceutical Sciences, Nitte (Deemed to be University), Deralakatte, Mangaluru 575018, India.; 6 Department of Applied Bioscience, College of Life and Environmental Science, Konkuk University, Seoul 05029, Republic of Korea.; 7 Department of Pharmacology, Saveetha Dental College and Hospitals, Saveetha Institute of Medical and Technical Sciences (SIMATS), Saveetha University, Chennai 600077, India.; 8 Department of Oral and Maxillofacial Surgery, Saveetha Dental College and Hospitals, Saveetha Institute of Medical and Technical Sciences, Saveetha University, Chennai 600077, India.

**Keywords:** *Pleurotus ostreatus*, Anthraquinone, MCF-7 cells, Matrix metallo-proteinases, CRISPR SpCas9

## Abstract

**Introduction**: Breast cancer results from tissue degradation caused by environmental and genetic factors that affect cells in the body. Matrix metalloproteinases, such as MMP-2 and MMP-9, are considered potential putative markers for tumor diagnosis in clinical validation due to their easy detection in body fluids. In addition, recent reports have suggested multiple roles for MMPs, rather than simply degeneration of the extracellular matrix, which comprises mobilizing growth factors and processing surface molecules.

**Methods**: In this study, the chemotherapeutic effects of anthraquinone (AQ) extracted from edible mushrooms (*Pleurotus ostreatus* Jacq. ex Fr.) cells was examined in MCF-7 breast cancer cells. The cytotoxic potential and oxidative stress induced by purified anthraquinone were assessed in MCF-7 cells using MTT and ROS estimation assays. Gelatin Zymography, and DNA fragmentation assays were performed to examine *MMP* expression and apoptotic induction in the MCF-7 cells treated with AQ. The genes crucial for mutations were examined, and the mutated RNA knockout plausibility was analyzed using the CRISPR spcas9 genome editing software.

**Results**: MCF-7 cells were attenuated in a concentration-dependent manner by the administration of AQ purified from *P. ostreatus* compared with the standard anticancer drug paclitaxel. AQ supplementation decreased oxidative stress and mitochondrial impairment in MCF-7 cells. Treatment with AQ and AQ with paclitaxel consistently decreased the expression of crucial marker genes such as *MMP2* and *MMP9*. The mutated genes *MMP2*, *MMP7*, and *MMP9* were assessed and observed to reveal four putative gene knockdown potentials for breast cancer treatment.

**Conclusions**: The synergistic application of AQ and paclitaxel exerted a strong inhibitory effect on the MCF-7 breast cancer cells. Extensive studies are imperative to better understand the action of bioactive mixes on the edible oyster fungus *P. ostreatus*. The gene knockout potential detected by CRISPR SpCas9 will aid in elite research into anticancer treatments.

## Introduction

Among the different types of cancer, breast cancer estimates at 2.26 million cases recorded in 2020, which is a considerable health challenge. Breast cancer is the primary cause of mortality in females 1. Breast cancer rates have increased significantly over the past two decades. Matrix metalloproteinases (MMPs) are a group of zinc metalloproteinases that act as a crucial function in degradation of extracellular matrix (ECM), angiogenesis, and remodeling. Current treatments for breast cancer such as chemotherapy have numerous side effects. Therefore, there is a need to identify novel, potent, and low-toxicity natural cancer treatments. MMP-2 and MMP-9 are associated with tumor growth, invasion, angiogenesis, and inflammation in breast cancer 2. Natural products, particularly those derived from plants and fungi, have become increasingly popular in cancer therapy owing to their high efficiency and fewer side effects. The edible mushroom *Pleurotus ostreatus* (PO) has received considerable attention owing to its potential therapeutic properties. It has been found to exhibit high efficacy against various cancers and degenerative diseases. Oyster mushrooms are widely cultivated edible mushrooms that possess medicinal properties such as hypocholesterolemia and antiatherogenic, antitumor, and antioxidant activities. Furthermore, crude oyster extract has been found to have pro-apoptotic and anti-proliferative effects on HT-29 cells and cytotoxic effects on PC3-cells 3,4. This article discusses the development of methods to validate natural drugs as anticancer agents. One such drug is anthraquinone (AQ), found in the edible mushroom PO, which has been analyzed for its anticancer activity against MCF7 breast cancer cells. AQ was extracted from PO and examined for its effect on cell viability and cytotoxicity in MCF7 breast cancer cells. The reactive oxygen species (ROS) induced by AQ were examined using a DCFH-DA fluorescence assay. Furthermore, we discuss the methods to evaluate the effectiveness of a natural anticancer agent: inhibition of collagen Type I synthesis and induction of apoptosis via the mitochondrial pathway. In addition, the downregulation of gelatin and collagenase MMP-2, MMP-7 and MMP-9 in the ECM of cells was validated using the CRISPR SpCas9 genome editing tool.

## Materials and Methodology

### Reagents

Anthraquinone extracted and purified as described by Bobek et al. 5. Human breast cancer cell line MCF-7 cells procured from NCCS Pune, Dulbecco's eagle modified medium (Sigma Aldrich Chennai), streptomycin (50 U/ml), penicillin (50 U/ml), 10 % fetal bovine serum (Sigma Aldrich Bangalore), Antibodies against MMP-2, MMP-9 were procured from Sigma Aldrich Chennai. Paclitaxel was procured from Dabur India, Ltd. Other chemicals and solvents were supplied by HiMedia (Mumbai, India).

### Cell culture

MCF-7 breast cancer cell lines were seeded in DMEM medium with 10% fetal bovine serum (FBS) supplemented with streptomycin (50 U/ml) and penicillin (50U/ml) at 37 °C and 50% CO_2_ and a relative humidity of 95%. The medium was replaced every three days. Monolayer cultures of MCF-7 cells were passaged at regular intervals using trypsin/EDTA (0.05/0.02%) in PBS without Mg^2+^ and Ca^2+^. The cells (2 × 10^5^) were plated, and the cell growth was observed to be exponential during 2-3 days in the medium. The cultured cells were kept with the test drug AQ at concentrations of 50-250 µg/ml, paclitaxel 0.001-10 µg/ml, and control cells were kept excluded with the drug.

### Cytotoxicity assay

The dose mediated impact of AQ on MCF-7 cell viability was analyzed with the trypan blue staining method (0.4%). The log phase cells was trypsinized and cultivated in cell culture plates (24 wells) at a rate of 5 × 10^4^ cells/ml in 1 ml of complete culture medium. The cells were kept with AQ and paclitaxel and the absorbance was noted at 570 nm using a microplate reader.



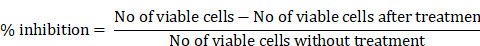



### Microscopical examination

The cells in the culture plate were examined under a light microscope for its morphological changes. The appearance of cells was noted at regular intervals, both in the presence and absence of drugs. This led to the confirmation of the effective concentrations of AQ (150 µg/ml) and paclitaxel (10 µg/ml). Further assays were performed using the aforementioned drug concentrations.

Experimental setup for *in vitro* analysis

The human breast cancer cells (MCF-7) were divided into 4 groups.

Group I: Normal control (no treated)

Group II: Paclitaxel (10 µg/ml)

Group III: AQ (150 µg/ml)

Group IV: Paclitaxel (10 µg/ml) + AQ (150 µg/ml)

### MTT Assay to determine the cell proliferation

MTT Assay was employed to analyse cell proliferation. The conversion of MTT-to-MTT formazan by mitochondrial enzymes was evaluated. Different concentrations of AQ and paclitaxel were added to the cells and incubated for 48 h. Before culturing the cells, 20 µl of MTT solution (5 mg/ml), 100 µl of 10% SDS and 0.01 N HCl was added to all wells and incubated for approximately 3h. After incubation, absorbance was measured at 570 nm.



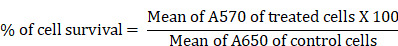



### Intracellular ROS measurement

Harvested cells were frozen and thawed in PBS (100 µl). The lysed cells were then centrifuged, and the supernatant was subjected to ROS quantification using an Oxiselect ROS assay kit (Cell Biolabs, USA). For intracellular ROS quantification, the centrifuged supernatant was stored with 10 µM DCFH-DA for 30 minutes. A fluorometer was used to measure the fluorescence intensity. The fold change was measured by dividing the fluorescence intensity of treated MCF-7 cells by that of untreated control cells.

### MMP potential analysis

The mitochondrial membrane potential (ΔΨ_M_) of MCF-7 cells was assessed by seeding the cells at a density of 1 X10^5^ cells/ml 6. The cells were incubated overnight. After incubation, the medium was restored with fresh DMEM added with 2% FBS and the drugs paclitaxel and AQ as per the grouping concentration. Later 24 h, the cells were trypsinized, washed with PBS, and added with 5',6',6'-tetrachloro-1,1'3',3'-tetraethylbenzimidazolyl carbocyanine iodide (10 µg/ml) for 20 min. Finally, the cells were washed with PBS, followed by centrifugation, the pellet was stored in fresh medium for flow cytometry analysis. The dye that accumulated within unscathed mitochondria was the dye that changed from red to green, which was due to membrane depolarization of mitochondria. The efficiency of the cells with green fluorescence was read as the depolarized mitochondrial membrane (ΔΨ_M_). The potential of AQ was compared to that of paclitaxel and untreated cells.

### Sub-G1 measurement by propidium iodide (PI) analysis

The 1 x10^5^ cells were treated with AQ and paclitaxel (Paclitaxel (10 µg/ml) + AQ (150 µg/ml) for 24 h. Subsequently, cells were spun, and the pelletized cells were mixed with 75% ethanol and stored overnight at -20 ^ᵒ^C 7. The cells were further spun and resuspended in PBS solution containing 1% PI (250 µl of PBS with 2.5 µl of PI) for 10 min in the dark at RT. The fluorescence intensity of approximately 10000 cells was measured by flow cytometry. The apoptotic index was then calculated. The readings were obtained from triplicate experiments.

### Annexin V staining

The 1 x10^5^ cells were treated with AQ and paclitaxel (Paclitaxel (10 µg/ml) + AQ (150 µg/ml) for 24 hrs. After the incubation period, the cells were centrifuged, and the pelletized cells were mixed with 75% ethanol and stored overnight at -20 ^ᵒ^C. Later, the cells were further spun and resuspended in PBS solution comprising 1% PI and Annexin V (250 µl of PBS with 2.5 µl of PI and Annexin V) for 10 min in the dark at RT. Around 10,000 cells were measured for its fluorescence intensity by flow cytometry. Besides, the apoptotic index was also calculated. Readings were taken from triplicate experiments performed.

### DNA fragmentation assay

The TUNEL assay is used to determine the fragmented DNA that results from apoptotic processes and specifically determined the nick in the DNA, which was further detected using terminal deoxynucleotidyl transferase (TdT). The MCF-7 cells were fixed with 2% paraformaldehyde, washed thrice using 0.1 M Tris buffer, fixed with absolute acetone for 1 min, washed with PBS, and incubated with AQ and paclitaxel (Paclitaxel (10 µg/ml) + AQ (150 µg/ml) at 37 ^ᵒ^C for 1 h. To which, 1.5 µM fluorescein isothiocyanate coupled with dUTP and TdT and approximately 10000 cells were analyzed using flow cytometry 8.

### Apoptotic assay by DAPI staining

The cells (3 × 10^5^) were plated and treated with paclitaxel and AQ (Paclitaxel (10 µg/ml) + AQ (150 µg/ml) overnight. The cells were then washed with PBS and fixed with 3.2% paraformaldehyde in PBS for 15 min at RT. The fixed and washed cells were stained with DAPI for 10 min at RT and washed again with PBS. Condensed cells with fragmented DNA were validated by fluorescence microscopy to detect the apoptosis ratio in MCF-7 cancer cells of the treated and untreated groups.

### Gelatin zymography assay

To evaluate MMP-2 and MMP-9 expression, MCF-7 cells were plated at a 3×10^5^ ratio. After washed with serum-free medium cells were kept for 24 h prior to treatment with AQ and paclitaxel. The treated media were subjected to SDS by mixing the cells with standard SDS gel loading dye comprising 0.01M SDS without β-mercaptoethanol. The soluble protein of MCF-7 cells was run in linear SDS PAGE and the electrophoresis for 6 h and the gels were washed with 100 ml of 2.5% triton X 100 at RT for 20 min. The gel was stained with CBB and destained using destaining solution.

### Statistical analysis

Statistical prediction was executed with Student's t-test software, and triplicate experiments were evaluated using two-way ANOVA. Data obtained from three independent experiments are expressed as the mean ± SD. P < 0.05 was kept as statistical significance.

## Results and Discussion

Cancer is a major fatal disease, and several efforts have been made to treat it using synthetic nanomaterials and naturally occurring compounds 9. This effort is urgently needed to overcome chemotherapeutic side effects and drug resistance. Etymological data support the concept that naturally occurring compounds with anticancer properties are safe for the human diet, nontoxic, and will definitely have long-term effects. Many studies have shown that the PO extract represses cancer cell proliferation by reducing cell differentiation. However, cytotoxicity varied significantly among the different samples. Many studies have validated the anticancer properties of mushrooms, such as *Agaricus* and *Pleurotus*. However, the molecular mechanisms underlying this phenomenon remain unclear. Thus, this study showed that the active compound observed in PO might be responsible for its anticancer activity.

### Cytotoxicity assay

**Figure 1:**
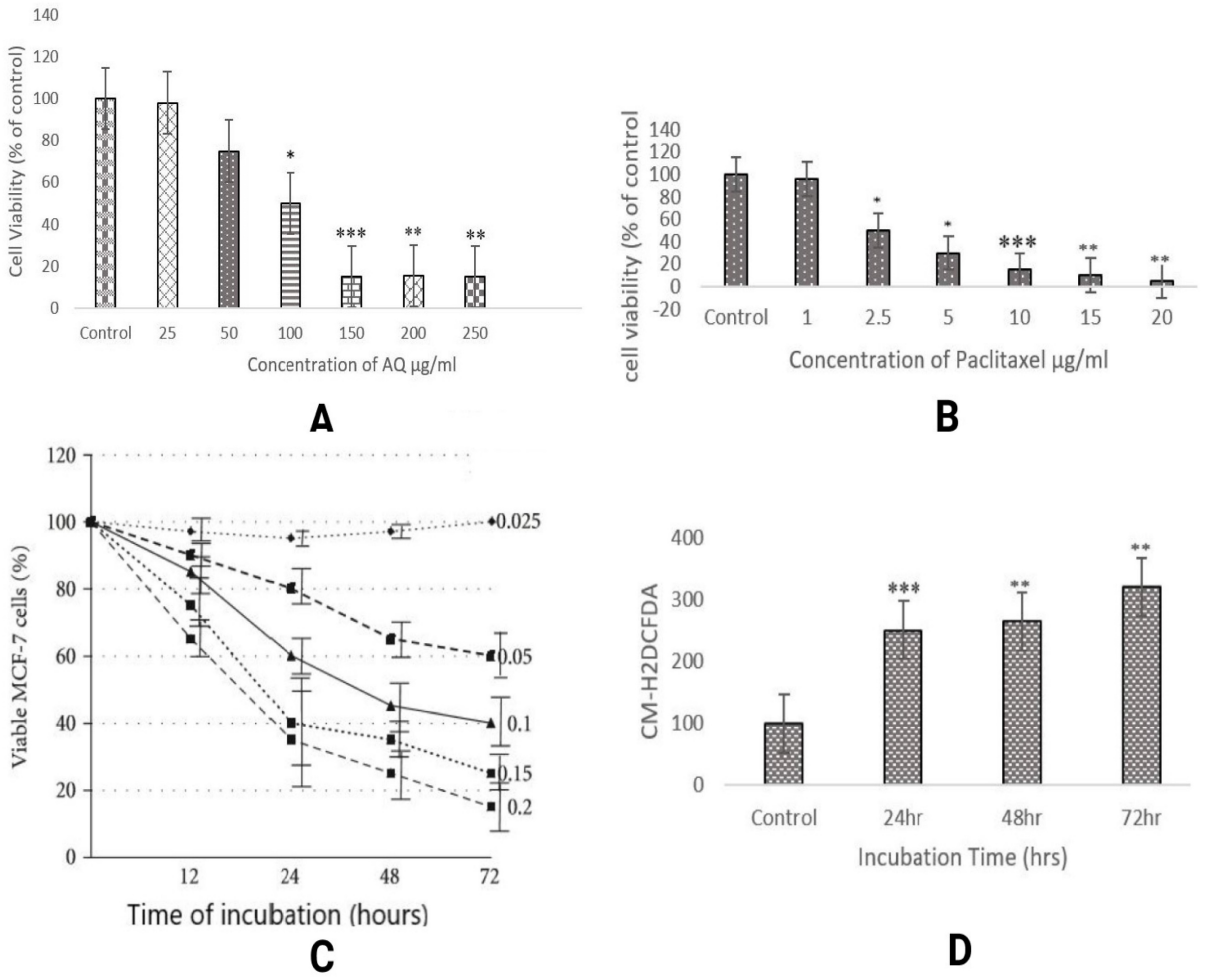
(**A**) Cytotoxicity of different concentrations of anthraquinone (25, 50, 100, 150, 200, and 250 µg/ml) in MCF-7 breast cancer cells was assessed at 570 nm. A significant difference between AQ-treated, AQ + paclitaxel, and control cells are shown at P < 0.05. (**B**) Cytotoxicity assessment of different concentrations of the paclitaxel standard anticancer drug (1, 2.5, 5,10, 15, and 20 µg/ml) in MCF-7 breast cancer cells was recorded at 570 nm. Significant difference between AQ treated, AQ + paclitaxel and control cells are showed at P < 0.05. (**C**) Cytotoxicity analysis with the MTT assay at varied levels of AQ (25, 50, 100, 150, 200, and 250 µg/ml) and paclitaxel standard anticancer drugs (1, 2.5, 5, 10, 15, and 20 µg/ml) in MCF-7 cells from a varying incubation period of 12 to 72 h was measured at 570 nm. Significant difference between AQ treated, AQ + paclitaxel and control cells are showed at P < 0.05. (**D**) Intracellular ROS assessment in MCF-7 cells: Positive Control: Graphical reading of intracellular images of MCF-7 cells without any treatment followed by AQ-treated cells under varying incubation periods of 24, 48, and 72 h.

It is one of the most vital parameters for *in vitro* evaluation of biological modifications. Even when drugs used *in vitro* are of natural origin, they exhibit different mechanisms of cytotoxicity against various cancer cells. Trypan blue staining executed in the study was performed to reveal viable or dead cells in suspension 10. It is a negatively charged large-sized molecule that works via the principle that living cells maintain a thick intact cell membrane that blocks the dye, whereas dead cells do not. MCF-7 cells were treated with AQ (25, 50, 100, 150, 200, and 250 µg/ml) and chemical anticancer drug paclitaxel (1, 2, 5, 5, and 10 µg/ml). The drug concentration of AQ was determined to be 150 µg/ml, and for paclitaxel, it was fixed at 10µg/ml based on the viability of the cells, as illustrated in Figure 1.

### MTT assay

MTT assay is a widely used colorimetric method to assess cytotoxicity and cell viability. This technique measures the activity of the mitochondrial enzyme, succinate dehydrogenase (SDH). In this process, MTT is converted to purple formazan by NADH based oxidoreductase enzyme 11. The intensity of this byproduct was determined by measuring the absorbance at 650 nm. To examine the effect of AQ on MCF-7 cell cytotoxicity, the cells were treated with concentrations ranging from 25 to 250 µg/ml for 24, 48, and 72 h, and the ratio of viable cells was analyzed. AQ decreased cell viability in a concentration-dependent manner; after 48 h, the IC50 value of AQ was 150 µg/ml (Figure 1). One of the predominant methods to determine the anticancer properties of naturally occurring agents is the MTT assay. A dose-dependent study was performed to examine the ratio of the drugs used for treating various cancers 12. This test can also be considered as a toxicity analysis of the extracted drugs. Different cell mortalities (p<0.05) were observed with varying levels of AQ at various incubation times. At various concentrations used, 150 µg/ml inhibited the cell growth to a maximum level of approximately 48 h. After increasing the incubation time to 72 h at a similar concentration, the cell viability was found to be stagnant at the same ratio of 150 µg/ml. Similar results were obtained by identifying the activity of mitochondrial enzymes 13.

### Intracellular ROS measurement

The results showed that AQ was cytotoxic to MCF-7 breast cancer cells, as estimated by the fluorescence intensity. The intracellular ROS levels in MCF-7 cells increased in a time-dependent manner from 24 h to 48 h to 72 h, indicating the potency of AQ in treating MCF breast cancer cells. Various studies have shown that ROS levels increase during hypoxia-reoxygenation conditions. An increase in ROS levels in the body can result in the upregulation of the invasiveness and motility of cancer cells. In this study, the advancement of migration and invasion of breast cancer cells was controlled by AQ treatment, which increased intracellular ROS levels, thereby activating the MAPK/ERK pathway. The level of intracellular ROS was significantly enhanced in MCF-7 cells after AQ treatment (Figure 1). Further studies revealed that ROS levels were significantly increased and inhibited cancer cells or induced apoptosis. ROS aggregation is associated with oxidative stress and is associated with cancer development. The mechanism employed to determine this was a cell-based assay to measure ROS activity. The pre-incubated MCF-7 cells with DCFH-DA are treated with AQ of different concentrations, and the incubated cells convert DCFH-DA by deacetylation by esterases in the cells to non-fluorescent DCFH. ROS in cells converts non-fluorescent DCFH to fluorescent DCF, which is further evaluated for its fluorescence intensity 14.

This fixed concentration was also supported by viable cells with a clear cytoplasm compared to the blue cytoplasm of dead cells. This is the best method for measuring cell membrane integrity. Based on the percentage inhibition, the dose-dependent effect of AQ on MCF-7 cell viability was analyzed. The colorimetric assay was performed at 570 nm using a microplate reader and the results were validated. Similarly, the AQ derivative 1,3-dihydroxy-9,10-anthraquinone-2- carboxylic acid (DHAQC) (2) and the natural AQ (damnacanthal) exhibited dose-dependent inhibition in the MCF-7 breast cancer cell line 15.

### Microscopical examination

To examine the modulatory effects and morphology of cancer cells after treatment with AQ and paclitaxel, microscopic examination was conducted. MCF-7 breast cancer cells grew flat in a star shape; however, after treatment with AQ, the cells were elongated and lifted, and most cells were widely spread.

**Figure 2:**
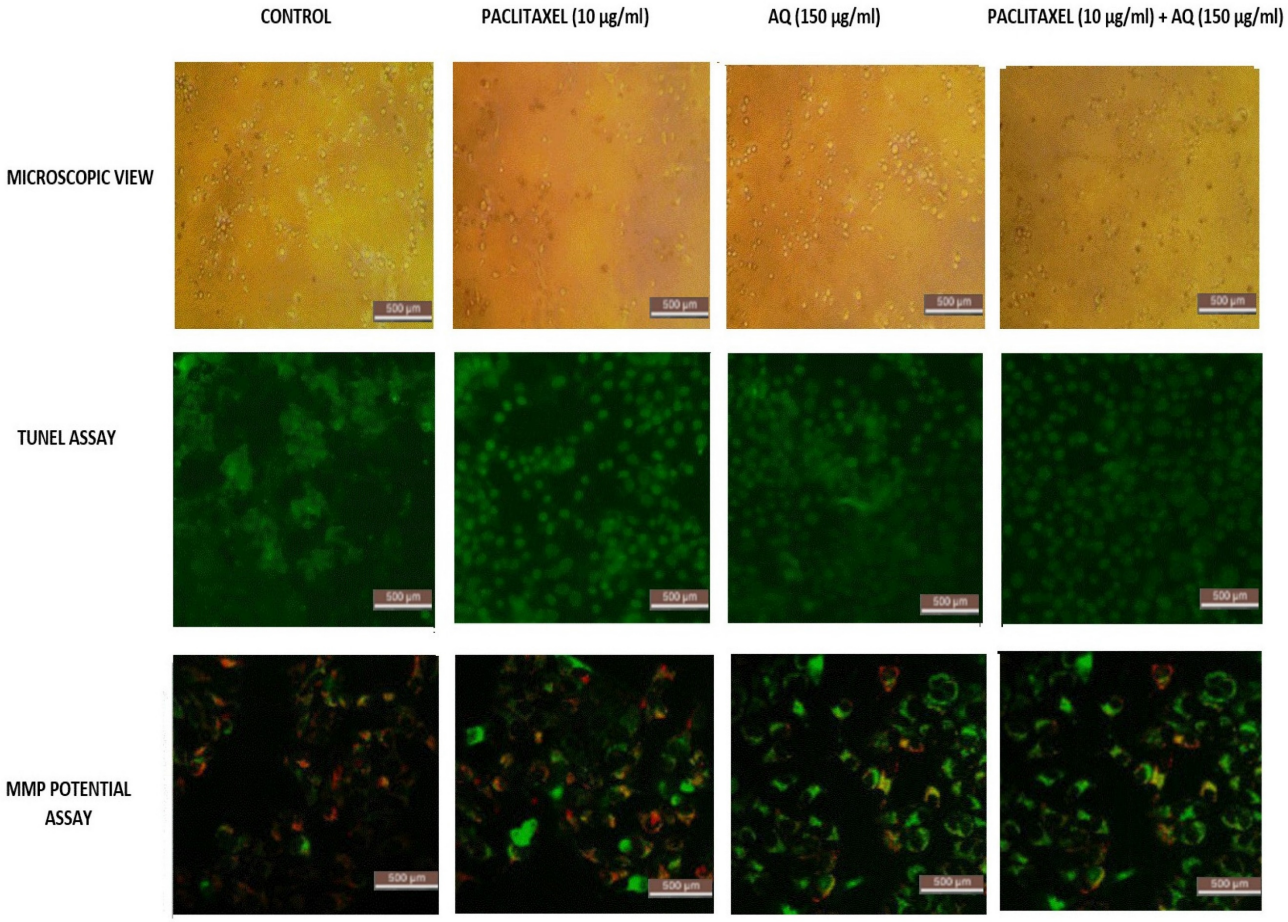
Microscopical assessment of MCF-7 breast cancer cells, TUNEL assay/DNA Fragmentation Assay and MMP activity: Positive control: Intracellular image of MCF-7 cells, Paclitaxel treated (10 µg/ml); AQ treated (250 µg/ml); AQ treated (250 µg/ml) + Paclitaxel treated (10 µg/ml); All images were taken with the same microscope settings.

Some cells were removed from adherence. Similar results were observed by Jedinak and Silva 16, who studied breast and colon cancer using *P. ostreatus*. As shown in Figure 2, the cell lines treated with 150 µg/ml showed a successive increase in cell viability over a period of 24 h. In fact, there is a catastrophe in the viability of the MCF-7 cancer cell lines. At 12 h, more than 75% of the MCF-7 cells retained cell viability as the incubation time increased to 24 h, and at 150 µg/ml, 95% of viable cells were lost, indicating that this is the ideal concentration to continue with other states pertaining to the anticancer activity of anthraquinone. The root extracts of AQ (xanthopurpurin) and 5 (lucidin-ω-methyl ether) from *Rubia philippinensis* showed significant toxicity against MCF7 and MDA-MB-231 breast cancer cell lines 17. The paclitaxel concentration used to treat MCF-cancer cells was fixed similarly based on the complete loss of viable cells at 10 µg/ml 18. Figure 2 clearly shows that if paclitaxel concentration is further increased, normal healthy cells may be damaged. Thus, the IC50 values for both AQ and paclitaxel were fixed.

### DNA fragmentation assay

This method was used to estimate the internucleosomal DNA cleavage. Cancer is one of the most common diseases in the world. Thus, a molecular understanding plays a vital role in drug delivery. Thus, the growing interest in programmed cell death over the last few years has led to an understanding of cell-cell mechanisms. DNA fragmentation assays are also known as DNA ladders or TUNEL assays. It depends on the TdT enzyme that adheres deoxynucleotides to the respective 3'-hydroxyterminus, where breaks in DNA are observed. Apoptosis was measured using biochemical markers (Figure 2). Compared with indirect methods, this direct nucleotide tagged with a fluorescent dye is the fastest. Thus, this method can be used as a biochemical marker for measuring apoptosis in cancer cells. The AQ-treated cells Figure 2 clearly showed inter-nucleosomal DNA damage, and fragmented DNA was clearly visible in MCF -7 cells after 24 h of incubation. Similarly, marine AQ (SZ-685C) revealed concentration-dependent DNA fragmentation in breast cancer cells, as indicated by the TUNEL assay 19.

### MMP potential analysis

The functional determination of mitochondria is a crucial factor in determining cell health. This has been well studied by analyzing the mitochondrial potential. Most of this energy is supplied by oxidative phosphorylation via the mitochondria, through which electron acceptors receive essential oxygen. The intensity of the dye increases when the cell membrane is highly damaged. The intensity of the generated emissions was noted. The reduction in ΔΨ_M_, as indicated by the fluorescence intensity, is shown in Figure 2. Figure 2 clearly shows that AQ significantly (p<0.01) depolarized the mitochondrial membrane of the cells after 24 h of treatment. The emission intensity was estimated to be 75% for MCF-7 cells. It is evident from the results that AQ treatment culminated in the interruption of ΔΨ_M_ in breast cancer cells 20. JC1 enters cancer cells and stains green because of loss of mitochondrial integrity. In addition, it is seen in the cytoplasm itself due to the entire collapse of membrane potential, whereas healthy cells allow the JC1 dye to enter the mitochondria via the cytoplasm and remain red. Thus, it is clear that for the MCF-cells exposed to AQ, the MMP increased significantly. These results suggest that AQ can enhance intracellular ROS levels, thereby inducing loss of viability in cancerous cells and promoting apoptosis in MCF-7cells. The anthraquinone quinazoline hybrid 7B showed significant mitochondrial impairment and increased apoptosis in MDA MB 231 cells 21.

Thus, these results support the morphological changes induced by AQ in PO. Morphological analysis is a preliminary indicator for determining the effect of anticancer agents, and thus, this will be an indicator for the use of various natural agents in treating cancer. Vibrant cell imaging with multiparametric analysis of images, such as the nucleus, cytoplasm, and culture contents, will help in designing drugs based on their efficiency by performing comparative analysis. In the future, drug modelling will be based on morphology-dependent features and color and texture levels from segmented images. Several anticancer studies on various mushrooms have indicated that morphology supports the efficiency in determining drug concentration [reviewed in 22].

### Propidium iodide staining to determine the sub G1

**Figure 3:**
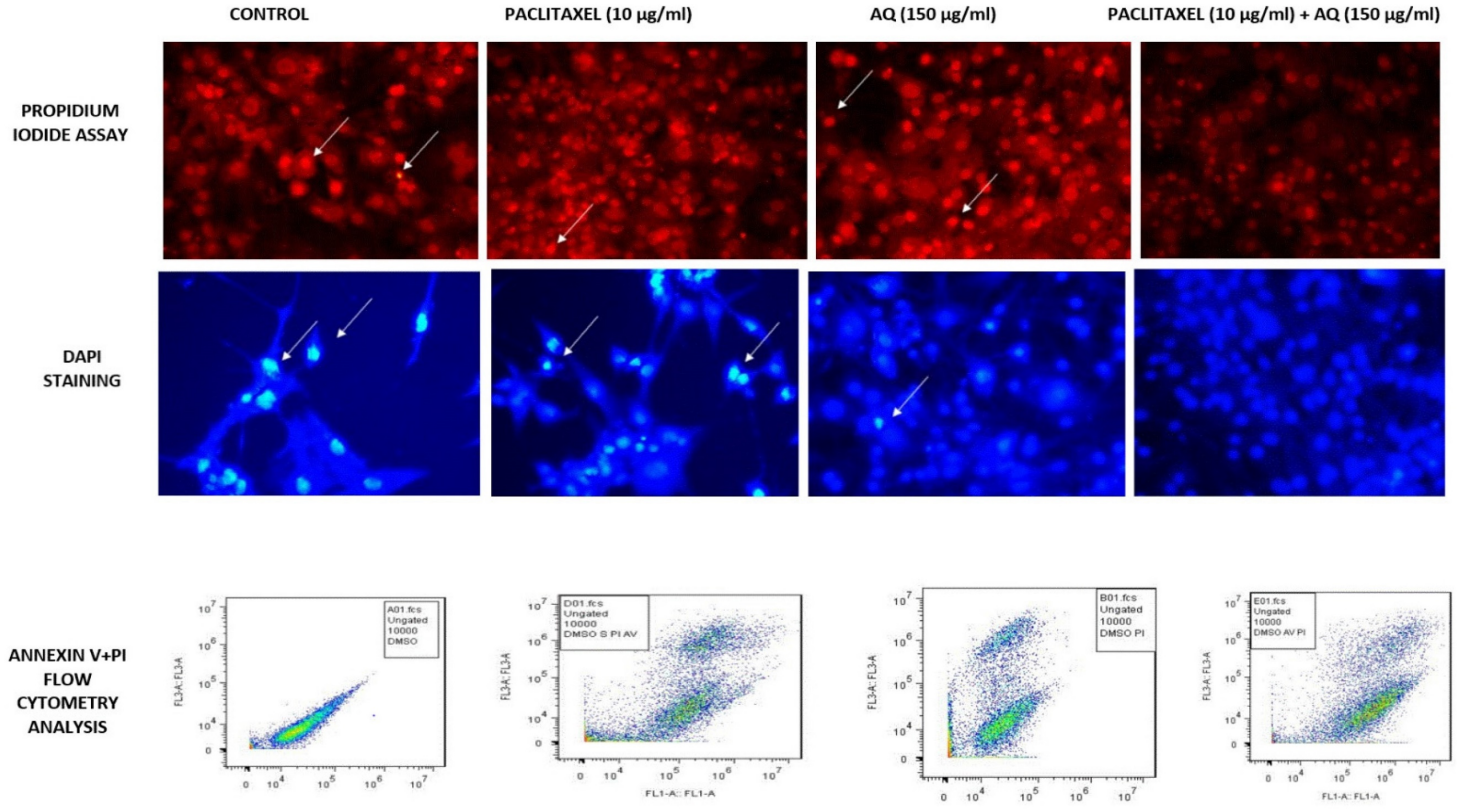
MCF-7 breast cancer cells stained with PI Staining, DAPI Staining, Annexin V and PI Staining: Positive Control: Intracellular image of MCF-7 breast cancer cells without treatment. Paclitaxel treated (10 µg/ml); AQ treated (250 µg/ml) reduces the mitochondrial dysfunction associated with apoptosis; AQ treated (250 µg/ml) + Paclitaxel treated (10 µg/ml) higher efficiency in reducing the mitochondrial dysfunction associated with apoptosis; All images were taken with the same microscope settings.

Cells stained with the fluorescent dye PI were used as intercalating dyes. It interacts with DNA, thus helping determine the effect of the drug on DNA. Thus, this method is widely used to determine apoptosis by estimating DNA fragmentation and nuclear content in cells. The results obtained after AQ treatment for 24 h and further incubation with PI revealed that AQ arrested the cell cycle and increased the ratio of cells in the G1 phase in MCF-7 cancer cells (Figure 3). From this study, it is clear that *P. ostreatus* might have active bioactive compounds such as AQ, which exhibit potency to inhibit the cell cycle in the G1 phase and promote apoptosis in MCF-7 cancer cells 16. DNA fragmentation in a dose-based manner was further observed in the percentage increase of cells in the subG1 phase when compared to control cells. The natural AQ aloin from Aloe showed cytotoxicity via enhanced apoptosis and G2M phase cell cycle arrest in breast cancer cell lines 23. To further confirm this assay, Annexin V staining and DNA fragmentation assays were performed (Figure 3).

### DAPI staining

DAPI is a fluorescent dye that binds alternatively to the AT-rich regions of dsDNA. Binding to these nucleotide regions resulted in a 20-fold increase in fluorescence intensity. From these results, it is clear that AQ treatment decreases the invasive ability of MCF-7 cells (Figure 3). This technique detects high levels of fluorescence in dead cells. Cancer cells lose permeability and membrane potential. Thus, the dye entered the cells very easily, enabling the fluorescence intensity to be very high, as determined by flow cytometry. The excitation wavelength was 340 nm, and the emission wavelengths were 488 nm and 460 nm. Blue emission by DAPI played a key role in determining apoptosis induced by AQ treatment in MCF-7 cells. Similar results were obtained for breast cancer cells treated with the PO extract stained with DAPI. The fluorescence intensity was found to be very high in a time- and dose-dependent manners 24.

### Annexin V staining

**Figure 4:**
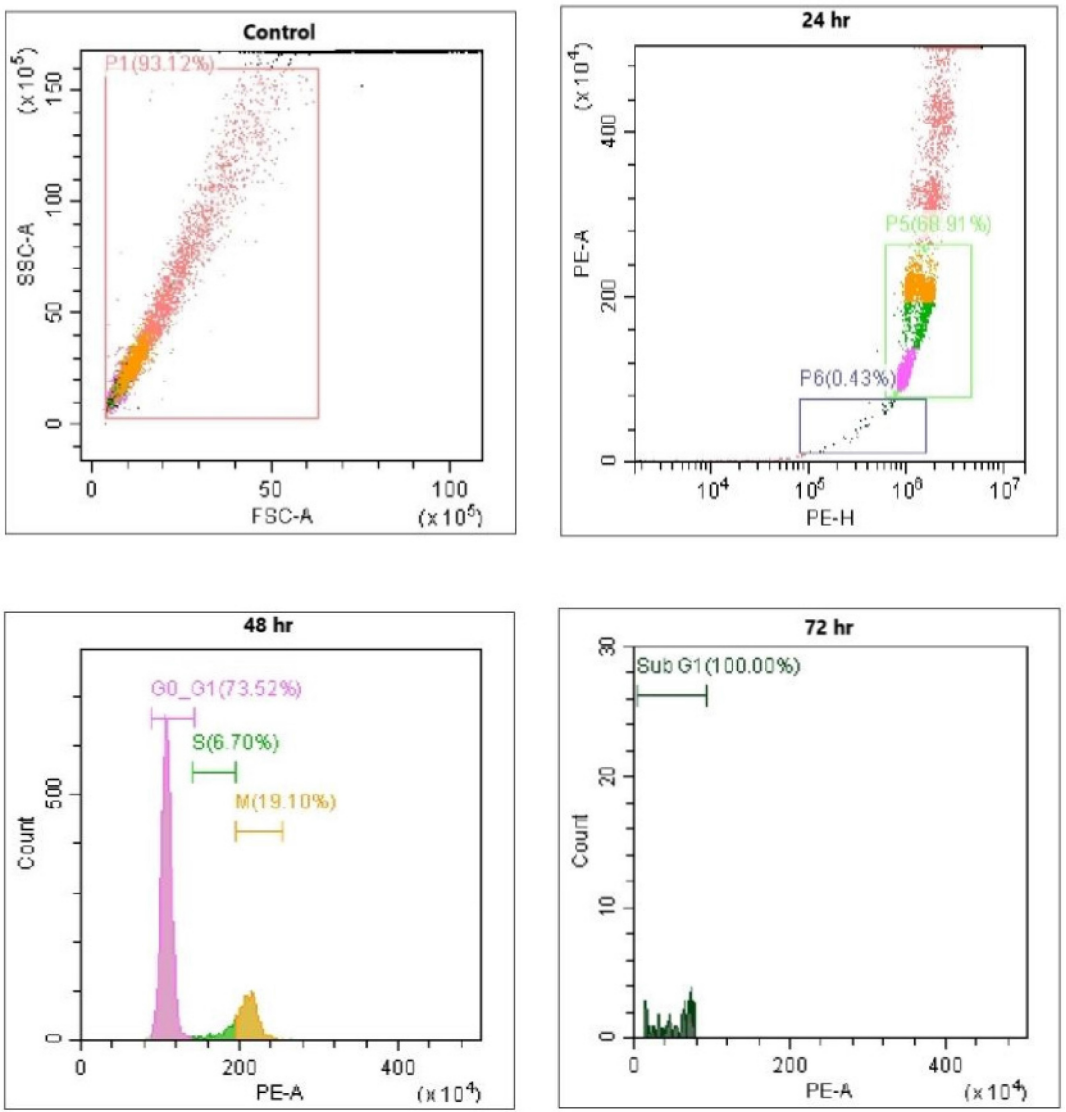
Evaluation of Flow cytometry in MCF-7 breast cancer cells conducted for PI and Annexin V staining to determine the sub G1 measurement of the cell cycle; Positive control: Flow cytometry image of MCF-7 cells without any treatment. Treatment with paclitaxel (10 µg/ml) and AQ (250 µg/ml) inhibited the cell cycle in G1 phase and promoted apoptosis; AQ treated (250 µg/ml) + Paclitaxel treated (10 µg/ml) enhanced efficiency in promoting apoptosis by inhibiting the cell cycle from G1 phase to S phase.

It is a phospholipid-binding protein that is a calcium-dependent factor that energetically binds to phosphatidylserine in the cell membrane. In normal cells, that is, noncancerous or healthy cells, the phosphatidyl serine layer is found on the inner side, which is impassable by annexin V. However, in cancerous cells, where permeability is already lost, annexin V dye easily enters (Figure 3). In addition, annexin V staining also determines apoptosis because the loss of permeability indicates that the cell is non-healthy, and phosphatidyl serine in the inner layer is already translocated, which is an irreversible process. These results indicated that the cells were in the stage of apoptosis and thus bound to annexin V dye. This was measured by the positive signal exhibited by the conjugation of Annexin V to FITC (Figure 3). Thus, the percentage of positive signals is directly correlated with the number of cells undergoing apoptosis, as indicated in Figure 4 25. Numerous studies have shown that *Pleurotus* spp. exhibit anticancer properties. Extracts from these mushrooms have been shown to induce increased apoptosis in breast cancer cells (MCF-7 and MDA-231), which was visualized by staining with Annexin V-FITC 13. Most studies that proved the same were conducted using only DAPI and TUNEL assays. Annexin and propidium iodide (PI) staining are considered to be the most important assays for determining cell permeability. Alteration of phosphatidylserine is a key factor in identifying the roles of apoptosis and phagocytosis. Incubation of cells with AQ followed by annexin V treatment resulted in a high proportion of apoptotic cells. The results obtained are in agreement with those of Abu et al. 26, who reported cell death caused by apoptosis in breast cancer cells (MDA-MB231 and MCF-7) following treatment with an anthraquinone (nordamnacantal) isolated from *Morinda citrifolia*.

### Gelatin zymography

**Figure 5:**
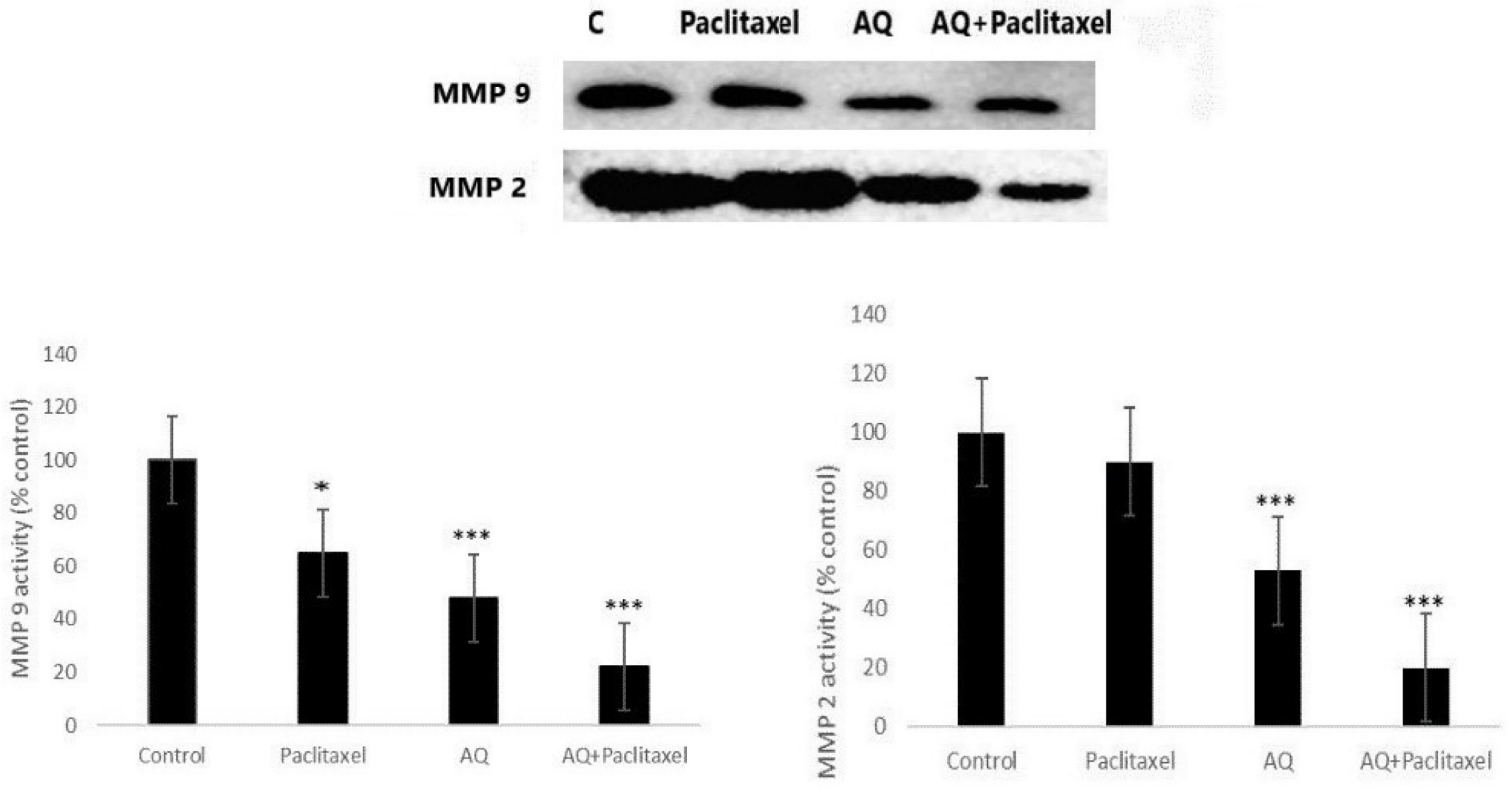
Gelatin Zymography analysis to measure the MMP2 and MMP 9 activity in positive control without any treatment; Paclitaxel treated (10 µg/ml); AQ treated (250 µg/ml); AQ treated (250 µg/ml) + Paclitaxel treated (10 µg/ml). Values are expressed as Mean ± SD of triplicate experiments. Significant difference between AQ treated, AQ + paclitaxel and control cells are indicated at P < 0.05.

This is a powerful yet simple method for determining proteolytic enzymes that are capable of derogating gelatin in cell signalling cascades. This test assay remains the key factor for assessing the two main matrix metalloproteinases (MMP-2 and MMP-9) because there are potent factors in degrading gelatin from biological sources. Thus, MMP-2 and MMP-9 were present in cells digested with gelatin attached to the polyacrylamide gel. After SDS, gels were transferred and stained with CBB. Therefore, degradation occurs when a location is visible against a deeply stained background. AQ was found to inhibit thrombolysis and tissue plasminogen activator (tPa)-induced MMP-9 and MMP-2 activities (Figure 5). It is evident from the graph that AQ prevented the activity of both MMP-2 and MMP-9. Thus, these proteolytic enzymes do not degrade gelatin within cells. AQ extract decreased tPA-tPA-inactivated MMP expression in MCF-7 cells. A similar test was performed to evaluate drugs in various cancers by determining the percentage of the active form of MMP-2 in tumor and normal tissue samples. MMP has been shown to influence prognosis via the invasion of lymph nodes and gastric system metastasis. MMP-9 expression may correlate well with tumor cell invasion and metastasis 27. MMP levels were highly expressed in invaded cells that were rapidly multiplying, which correlated with the harmful and malignant characteristics of tumor cells. The assay for determining MMP was an independent prognostic factor. In line with this, the PO ethanolic extract reduced the expression of MMP-9, and molecular docking analysis showed that lovastatin, the compound identified from the extract, showed enhanced binding with either MMP-2 or MMP-9 28. Furthermore, the polysaccharide extract PO exhibited a potent reduction in the secretion of MMP-2 and 9 that indicates a reduction in colon cancer cell invasion 3.

#### Gene knockout analysis

Gene knockout possibilities in treating cancer using matrix metalloproteinases were analyzed using the CRISPR spcas9 tool, and the four best gene knockout possibilities in treating cancer and their respective RNA sequences with the chromosome number in a human being were identified. Table S1 shows the knockout sequences of *MMP-2*, *MMP-7* and *MMP-9*. Figure S1 (A-E) shows the exact position of the sequence. A similar analysis was performed for the lung cancer treatment. Viral-mediated delivery of drugs by optimizing genes using CRISPR is a recent technology used to treat cancer 29. Thus, these gene knockout sequences will pave the way for the use of therapeutic drugs to treat various cancers 30-32.

## Conclusions

From this study, it is evident that the edible fungus *P. ostreatus* and its active bioactive compound AQ can be potent anticancer agents for the treatment of breast cancer cells by triggering apoptosis through depolarization of the mitochondrial membrane. Furthermore, other assays, such as cytotoxicity, intracellular ROS, Sub G1 measurement, G1/S checkpoint analysis, Gelatin Zymography and TUNEL assay also validated that AQ-treated MCF-7 cells showed a high degeneracy rate. These assays were conducted *in vitro* to validate the efficacy and potency of AQ. Therefore, clinical trials using an *in vivo* approach are necessary. Furthermore, the gene knockout mechanism of *MMP-2*, *MMP-7*, and *MMP-9* shown by CRISPR-Sp cas9 to improve cancer treatment at the gene level will also pave the way for future approaches in the treatment of breast cancer. Detailed comprehensive studies employing the *in vivo* and clinical studies are essential to understand the mechanism of action of AQ derived from *P. ostreatus*.

## Supplementary Material

Supplementary figure and table.
