# Supplementary material for: Evaluation of oyster mushroom (Pleurotus ostreatus)-derived anthraquinone on the induction of apoptosis and suppression of MMP-2 and MMP-9 expression in breast cancer cells
Source: Int J Med Sci. 2024 Apr 8;21(6):1016–26. doi: 10.7150/ijms.93334 (PMC11103395; doi:10.7150/ijms.93334)
Supplement: Supplementary file 1 — Supplementary figure and table. [file ijmsv21p1016s1.pdf]

Supplementary Materials

Supplementary Table:

Table S1: Gene sequence analysis possibilities for treating breast cancer via editing *MMP-2, MMP-7* and *MMP-9* RNA sequences using CRISPR SpCas9 Genome editing tool.

Gene sequence analysis	**MMP 2**	**MMP 7**	**MMP 9**
Guide RNA Sequence	UAAGGUCACCGUUGCAGGCC	GGGCCGCGGCGCCACCAUGG	AAAGAGCAAGUGACAAAUGU
Gene Knockout Sequence	TAAGGTCACCGTTGCAGGCC	GGGCCGCGGCGCCACCATGG	TGTAAACAGTGAACGAGAAA
Guide RNA Sequence	GUCACCGUUGCAGGCCUGGC	CGCCUGUCGCACCGCCAUGG	GAGCAAGUGACAAAUGUUGG
Gene Knockout Sequence	GTCACCGTTGCAGGCCTGGC	GGUACCGCCACGCUGUCCGC	GGTTGTAAACAGTGAACGAG
Guide RNA Sequence	GUACUGGGUCUUUUCCAGCC	CAGCGCCUGUCGCACCGCCA	GACAAAUGUUGGAGGAGCAG
Gene Knockout Sequence	CCGACCUUUUCUGGGGUCAUG	ACCGCCACGCUGUCCGCGAC	GACGAGGAGGTTGTAAACAG
Guide RNA Sequence	CAGGACAAAUGACCACAUCA	CGCCACCAUGGCGGUGCGAC	UGUUGGAGGAGCAGUGGUGA
Gene Knockout Sequence	ACUACACCAGUAAACAGGAC	CGCCACCATGGCGGTGCGAC	AGTGGTGACGAGGAGGTTGT

Supplementary figures:

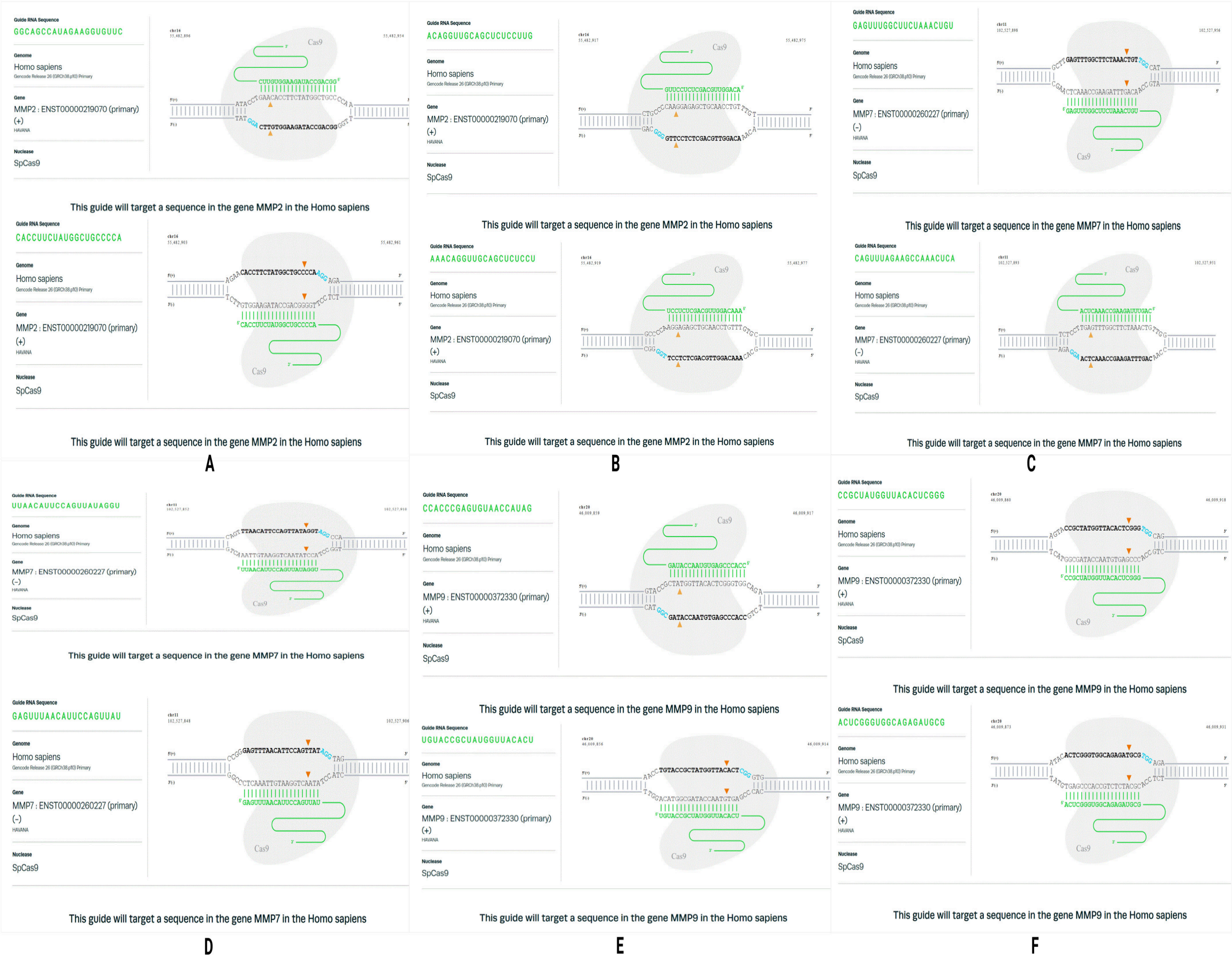

Figure S1. A: Gene knockout possibilities predicted by the CRISPR Spcas9 genome editing tool for *MMP-2*-Target sequence-1 5'GGCAGCCAUAGAAGGUGUUC3' & Target sequence 2- 5'CACCUUCUAUGGCUGCCCCA3'in *Homo sapiens*.

B: Gene knockout possibilities predicted by the CRISPR Spcas9 genome editing tool for *MMP-2*-Target sequence-3 5'ACAGGUUGCAGUCUCCUUG3' & Target sequence 4- 5'AAACAG GUUGCAGCUCUCCU3'in *Homo sapiens*.

C: Gene knockout possibilities predicted by the CRISPR Spcas9 genome editing tool for *MMP-7*-Target sequence 1 5'GAGUUUGGCUUCUAAACUGU3' & Target sequence 2- 5'CAGUUUAGAAGCCAAACUCA 3'in *Homo sapiens*.

D: Gene knockout possibilities predicted by the CRISPR Spcas9 genome editing tool for *MMP-7*-Target sequence-3 1 5'UUAACAUUCCAGUUAUAGGU3' & Target sequence 4- 5'GAGUUUAACAUUCCAGUUAU3'in *Homo sapiens*.

E: Gene knockout possibilities predicted by the CRISPR Spcas9 genome editing tool for *MMP-9*-Target sequence-1 5'CCACCCGAGUAUAACC' & Target sequence 2- 5'UGUA CCGCUAUGGUUACACU3'in *Homo sapiens*.

F: Gene knockout possibilities predicted by the CRISPR Spcas9 genome editing tool for *MMP-9*-Target sequence-1 5'CCGCUAUGGUUACACUCGGA3' & Target sequence 2-5'ACUCGGGUGGCAGAGAUGCG3'in *Homo sapiens.*
